# Genotypic and Phenotypic Profile of 50 Cases With Chromatin Remodeling Complexes‐Related Neurological Disorders

**DOI:** 10.1002/cns.71061

**Published:** 2026-07-29

**Authors:** Shimeng Chen, Fei Yin, Fang He, Ciliu Zhang, Xiaolu Deng, Lifen Yang, Li Yang, Chen Chen, Fangyun Liu, Jing Peng

**Affiliations:** ^1^ Department of Pediatrics Xiangya Hospital of Central South University Changsha China; ^2^ Clinical Research Center for Children Neurodevelopmental Disabilities of Hunan Province Changsha China

**Keywords:** chromatin remodeling complexes, epilepsy, GDD/ID, genotype, phenotype

## Abstract

**Aim:**

To describe the genotypic and phenotypic profile of chromatin remodeling complex (CRC)‐related neurological disorders.

**Methods:**

We retrospectively analyzed the clinical characteristics and the genetic spectrum of children with variants in CRC genes.

**Results:**

Fifty patients were included. The genotype spectrum involved genes encoding the CHD complex (*CHD2*, *CHD3*, *CHD4*, *CHD7*, *CHD8*), the BAF complex (*SMARCA2*, *ARID1B*, *ARID2*, *ACTB*), and the ISWI complex (*BPTF*). The predominant phenotypes were global developmental delay/intellectual disability (GDD/ID) (76.0%, 38/50) and epilepsy (64.0%, 32/50). Seventy‐five percent of patients with epilepsy had generalized seizures. Sixty‐nine percent (20/29) were seizure‐free for more than 1 year at the last follow‐up (three were lost to follow‐up), although 56.3% (18/32) of patients required three or more kinds of antiseizure medications. EEG abnormalities were prevalent (82.2%, 37/45), commonly showing generalized or diffuse interictal discharges (63.6%), slow background activity (59.5%), and slow waves (35.1%). BAF complex variants were significantly associated with GDD/ID, microcephaly, facial dysmorphism, short stature, and slow background activity on EEG, while CHD complex variants were more likely to cause heat‐sensitive seizures (FDR < 0.05).

**Conclusion:**

CRC‐related disorders are primarily caused by variants in the CHD and BAF complexes, with *CHD2*, *ARID1B*, and *SMARCA2* being the frequently involved genes. The conditions are characterized by GDD/ID and epilepsy, with distinct clinical patterns between the CHD and BAF complexes.

## Graphical abstract

CRC‐related neurological disorders are mainly caused by variants in the CHD and BAF complex. The predominant phenotypes of CRC‐related neurological disorders were GDD/ID and epilepsy. Variants in the CHD and BAF complexes have different phenotypes.

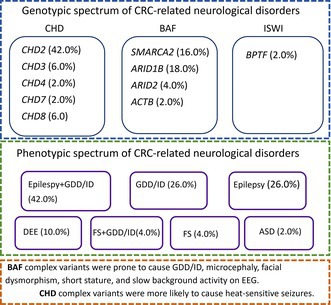

## 1 Background

Chromatin remodeling complexes (CRCs) have essential roles in the ATP‐dependent chromatin remodeling process that disrupts nucleosome DNA contacts, moves nucleosomes along the DNA, and removes or exchanges nucleosomes, making sure DNA is accessible for transcription, replication, recombination, as well as repair [1]. There are four large chromatin remodeling complexes (CRCs) families depending on their central ATPase subunit: BAF (Brg1, hBrm), originally known as the SWI/SNF complex, INO80/SWR1 (hINO80, hDomino, SRCAP), ISWI or NURF (hSNF2H, hSNF2L), and CHD or NuRD (CHD1‐9) [2]. CRCs are widely studied in cancer and have been known to play a part in neuroscience, such as neuronal development and synaptic plasticity.

Genes encoding CRCs are reported to be implicated in childhood neurological diseases, such as epilepsy, intellectual disability (ID) or global developmental delay (GDD), and autism spectrum disorder (ASD). Chromodomain‐helicase‐DNA‐binding (CHD) proteins have two histone tail‐binding domains, a central and conserved SNF2‐like helicase motif, and a C‐terminal DNA‐binding domain [3]. There are nine members (*CHD1*–*CHD9*) in the CHD family, and *CHD2*, *CHD3*, *CHD4*, *CHD5*, *CHD6*, *CHD7*, and *CHD8* have phenotypes of epilepsy, ASD, and intellectual disability (ID) [1, 4]. The ISWI complex is composed of one ATPase subunit and one to three noncatalytic subunits. Nearly 16 genes are involved in the ISWI complexes, with all regulatory subunits taken into account. Most genes play roles in cancer, while *BPTF* is implicated in neurodevelopmental disorders (NDDs) [5, 6]. The BAF complex and INO80/SWR1 complex have multiple subunits. Mammalian BAF complexes are an assembly of at least 15 subunits encoded by 29 genes [7], among which *ARID1A*, *ARID1B*, *ARID2*, *SMARCA2*, *ACTB*, *SMARCA4*, *SMARCB1*, *SMARCC1*, *SMARCC2*, and *PBRM* were reported to be related to neurodevelopmental and psychiatric disorders [2, 7, 8, 9]. The INO80/SWR1 complex is composed of more than 15 subunits, and not less than 20 genes are contained [10, 11]. Variants in genes in this complex are reported to cause NDDs, too [12].

Here, we reported the genotype and phenotype spectrum of CRC‐related neurological disorders enrolled in a single pediatric neurology center consisting of 50 patients.

## 2 Methods

### 2.1 Participants and Grouping

From January 2015 to 2025, we recruited 50 subjects aged less than 18 years who presented with neurological disorders with variants in CRCs. The diagnostic decisions were made by neurologists in the Pediatric Department of Xiangya Hospital. DSM‐5 criteria for GDD/ID, attention deficit and hyperactivity disorders (ADHD), and ASD (Diagnostic and Statistical Manual of Mental Disorders, Fifth Edition, American Psychiatric Association, 2013), and the International League Against Epilepsy (ILAE) definitions of seizures, epilepsy, and epileptic syndromes, as well as developmental epileptic encephalopathy (DEE), were followed [13, 14]. Demographics and clinical data were reviewed according to our medical records. The progression of epilepsy and febrile seizures (FS) was given close attention. The results of auxiliary examinations, mainly cranial magnetic resonance imaging (MRI) and electroencephalogram (EEG), were also re‐evaluated. Forty‐nine patients were divided into two groups based on the causative genes to compare the differences in phenotype: the CHD group, 29 patients who had variants in the CHD complex; and the BAF group, 20 patients who had variants in the BAF complex. One patient with a variant of the ISWI complex was not grouped owing to the insufficient sample size.

### 2.2 DNA Tests, Data Analysis, and Clinical Assessment

Most patients (47/50) opted for whole‐exome sequencing (WES), and three patients had clinical whole‐exome sequencing or gene panel sequencing. The methods for sequencing and genetic testing have been described in detail in our previous study, and variants were analyzed and classified based on the American College of Medical Genetics (ACMG) guidelines and clinical assessment as we used to [15, 16]. Only pathogenic or likely pathogenic variants were included in this study.

### 2.3 Statistics

This study used descriptive statistical analysis to summarize the baseline demographic and clinical phenotypic characteristics of enrolled participants. Categorical variables were presented as counts and corresponding percentages [*n* (%)], while continuous variables that did not follow a normal distribution were described using the median and interquartile range (IQR). The clinical characteristics between the CHD and BAF complexes were compared via Fisher's exact test. Multiple comparisons were adjusted for the false discovery rate (FDR) using the Benjamini–Hochberg (BH) method to reduce the risk of inflated type I errors, and differences were considered statistically significant if the adjusted FDR was less than 0.05. All quantitative statistical analyses and data processing were performed using SPSS version 20. Additionally, before multiple testing correction, an initial threshold of *p* < 0.05 was used to indicate potential statistical significance.

## 3 Results

### 3.1 Demographic Features

This study included 50 patients, aged from 2 to 17 years, with a median age of 9 years (IQR, 6–12). One patient passed away at 6 years old. The sex ratio was 1.3:1 (male vs. female). Patients one and two were siblings from a family, and patients 30 and 31 were brothers. The remaining 46 were isolated subjects.

### 3.2 Genetic Testing Results

**FIGURE 1:**
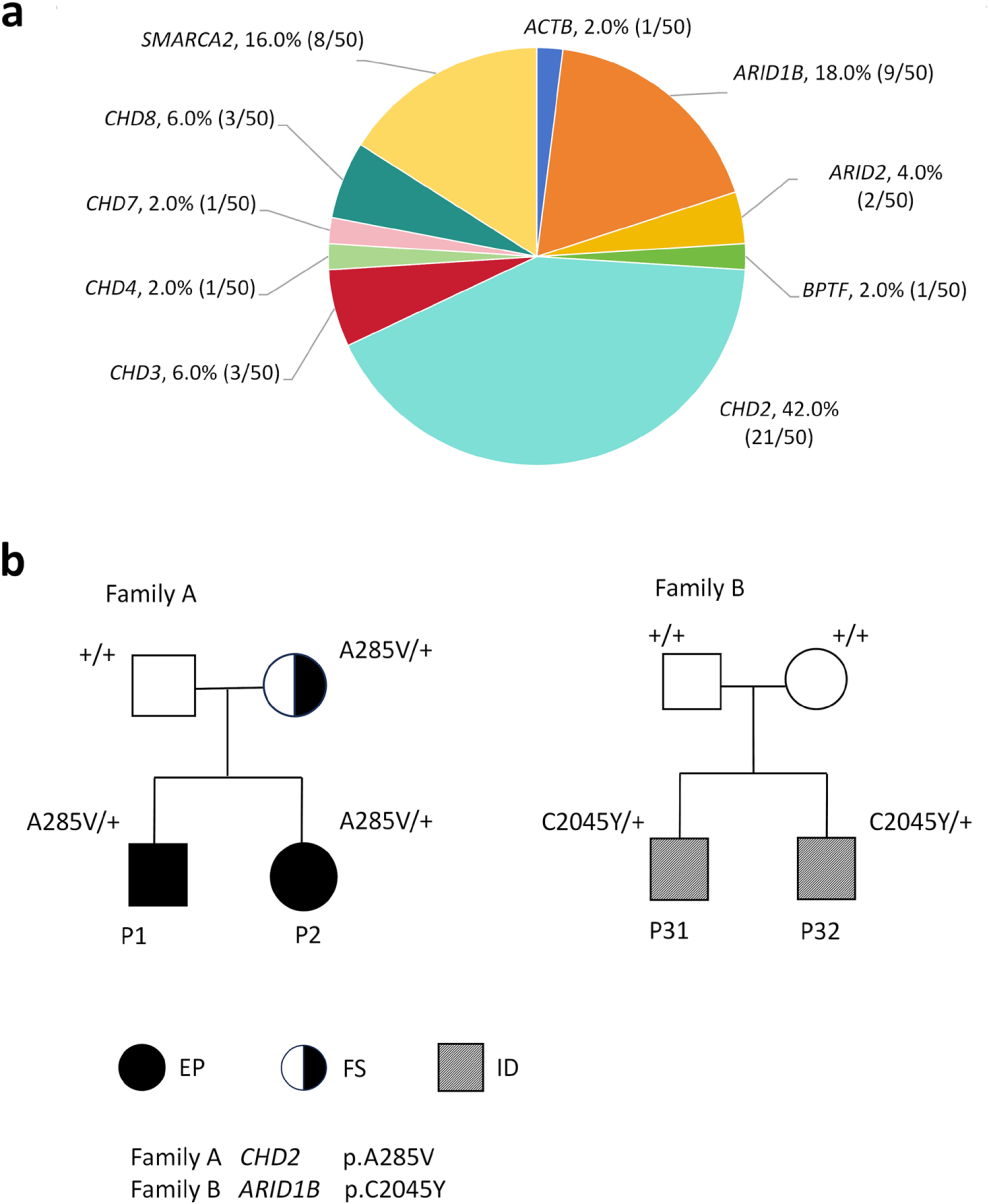
Composition of 50 CRC‐related neurological disorders and pedigree of two families in this study. (a) Genes of CRC included in this study. (b) Pedigree charts of family A and B.

Fifty variants in five genes encoding the CHD complex (*CHD2*, *CHD3*, *CHD4*, *CHD7*, and *CHD8*), four genes encoding the BAF complex (*ARID1B*, *SMARCA2*, *ARID2*, and *ACTB*), and one gene involved in the ISWI complex (*BPTF*) were detected. Variants in *CHD2* accounted for 42.0% (21/50), and variants in *ARID1B* and *SMARCA2* were also common, constituting 18.0% (9/50) and 16.0% (8/50), respectively. Variants in *CHD4*, *CHD7*, *ACTB*, and *BPTF* were less common, being found in one individual patient (Figure 1a). Two variants in *SMARCA2*, p.R855Q and p.E852K, were found in two unrelated cases independently. Two families were enrolled in this study. Family A carried the variant p.A285V of *CHD2*, and family B had the variant p.C2045Y of *ARID1B* (Figure 1b). In total, 46 different variants were reported in this context, and 28 variants have not been reported previously in the literature or ClinVar (detailed information can be seen in the additional file Table S1).

**FIGURE 2:**
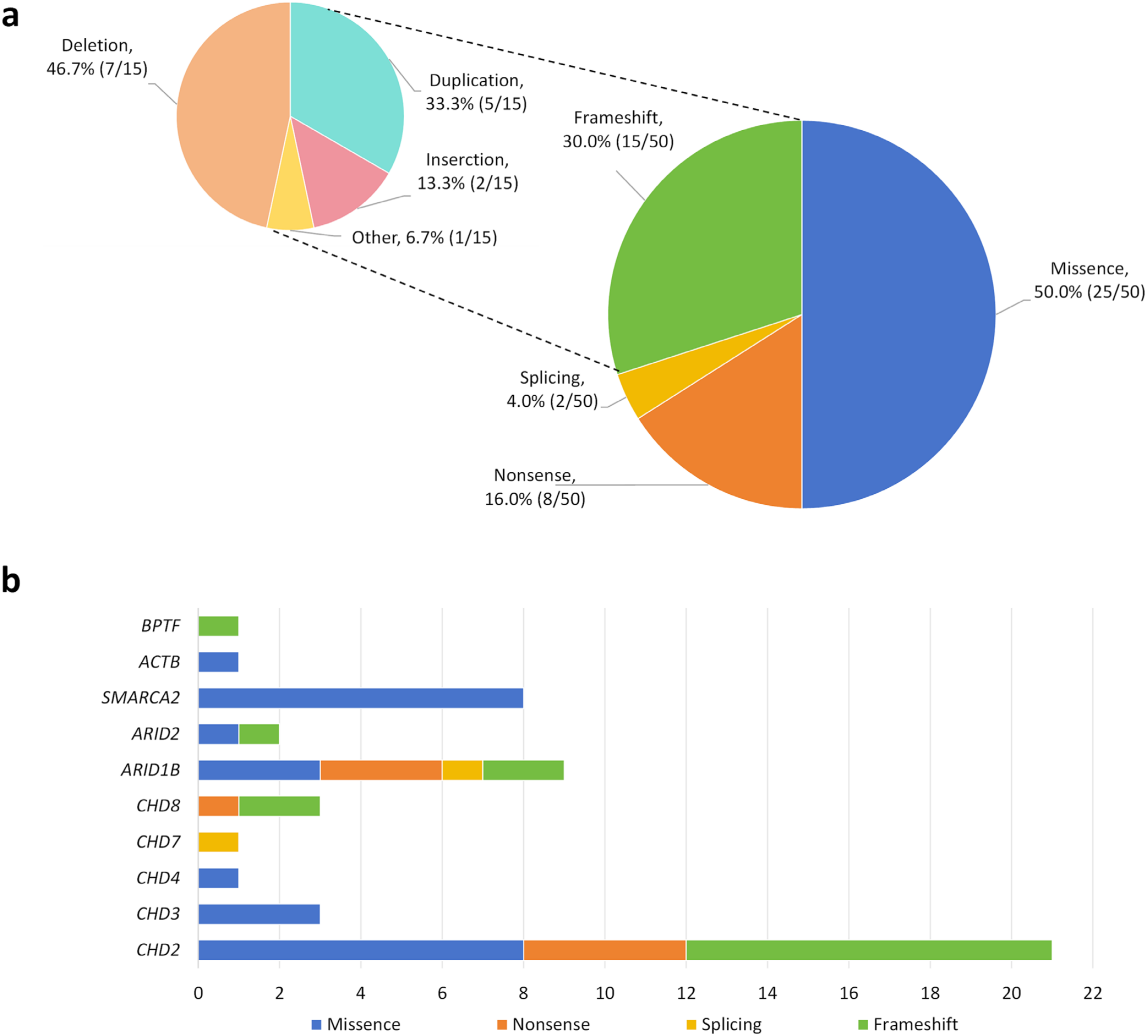
Types of variants in 50 patients. (a) The overall types of 50 variants. (b) Variant types of different genes.

Missense variants made up half of those CRC variants (25/50, 50.0%), followed by frameshift (15/50, 30.0%). Nonsense variants and splice site variants accounted for 16.0% and 4.0%, respectively (Figure 2a). Deletion (7/15), duplication (5/15), insertion (2/15), and single‐nucleotide variants (1/15) contributed to frameshift variants. In the CHD complex group, 58.6% of variants (17/29) were likely gene‐disrupting (LGD) variants (nonsense, frameshift, and splice site). In contrast, 65% of variants (13/20) in the BAF group were missense (Figure 2b).

Most variants (45/50, 90.0%) were de novo. Three variants in *CHD2* were inherited from a mildly affected mother, and two variants in *CHD8* were passed from an unaffected parent. All variants followed the autosomal dominant inheritance pattern.

### 3.3 Clinical Characteristics and Diagnosis

**FIGURE 3:**
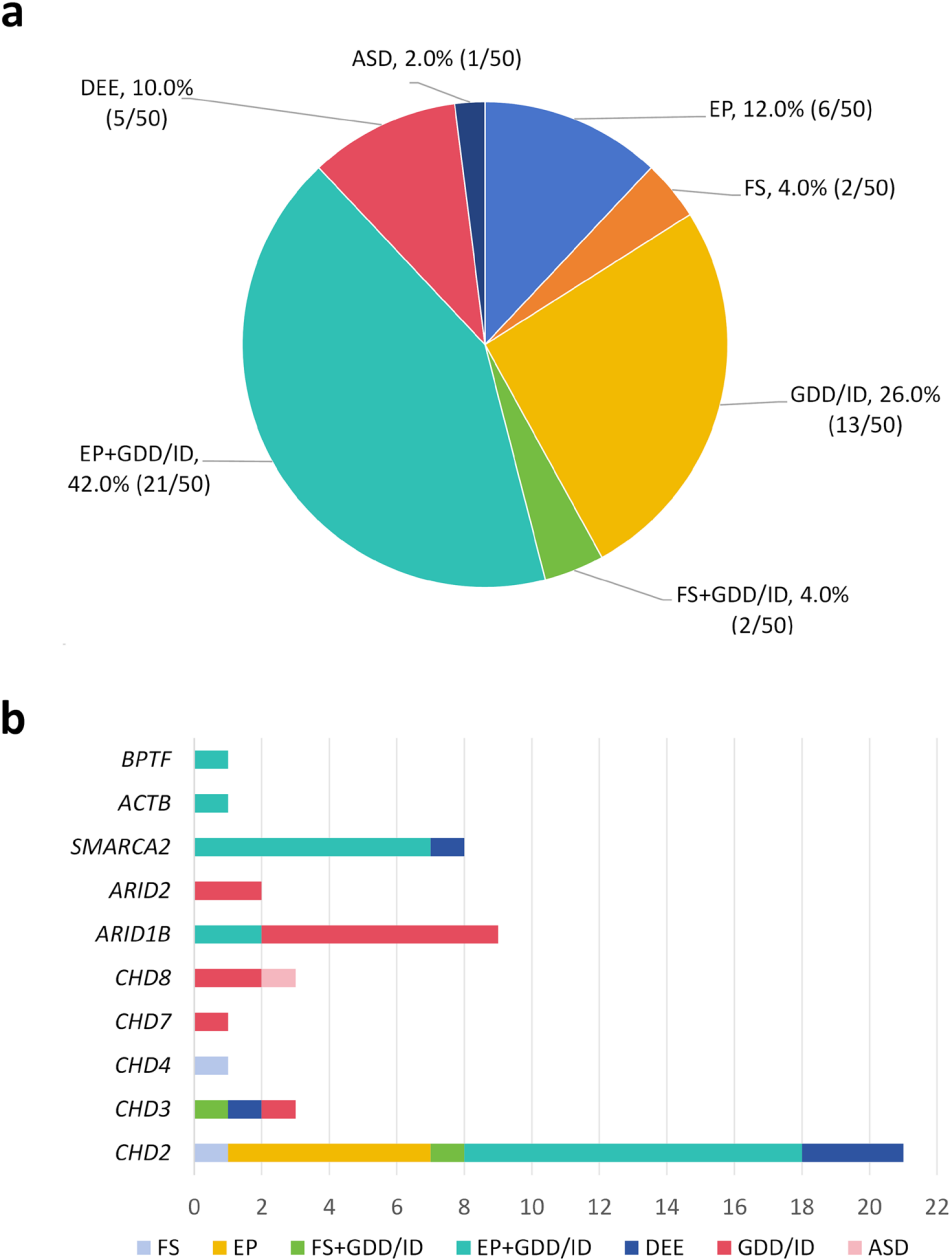
Main clinical diagnosis of 50 patients. (a) The overall clinical diagnosis of 50 patients. (b) Clinical diagnosis of different genes.

GDD/ID and epilepsy were the top two common phenotypes of this cohort. Figure 3a showed the main clinical diagnosis of those 50 patients. GDD/ID comorbid with epilepsy was diagnosed in 42.0% (21/50) of patients. Isolated GDD/ID and isolated epilepsy were diagnosed in 26.0% (13/50) and 12.0% (6/50) cases, respectively. Five cases were diagnosed as DEE (5/50, 10.0%), including infantile epileptic spasm syndrome (IESS), Lennox–Gastaut syndrome, Doose syndrome, and Dravet syndrome. GDD/ID with FS, isolated FS, and ASD were diagnosed in several cases. The diagnoses of patients with different gene variants are displayed in Figure 3b. The diagnosis of CHD complex variants was diverse; for example, diagnoses for *CHD2* varied from FS to GDD/ID comorbid with epilepsy and DEE. The diagnosis of BAF complex variants was relatively restricted, mainly GDD/ID or GDD/ID comorbid with epilepsy. Other clinical features, such as behavior problems, microcephaly, short stature, and facial dysmorphism, were also observed (Table 1).

**TABLE 1:** Phenotype in 50 patients with CRC variants.

Characteristics	*N* (%)	Genes (No. of patients)
Epilepsy	32/50 (64.0%)	/
Focal	7/32 (21.9%)	*CHD2* (3), *ARID1B* (2), *SMARCA2* (1), *ACTB* (1)
Generalized	19/32 (59.4%)	*CHD2* (13), *CHD3* (1), *SMARCA2* (5)
Combined	5/32 (15.6%)	*CHD2* (2), *SMARCA2* (2), *BPTF* (1)
Unknown	1/32 (3.1%)	*CHD2* (1)
Reflex seizures	3/32 (9.4%)	*CHD2* (3)
Photosensitive	2	*CHD2* (2)
Sound‐sensitive	1	*CHD2* (1)
Heat‐sensitive seizures	5/32 (15.6%)	*CHD2* (4), *ACTB* (1)
DEE	5/32 (15.6%)	*CHD2* (3), *CHD3* (1), *SMARCA2* (1)
Infantile epileptic spasm syndrome	1	*CHD3* (1)
Lennox–Gastaut syndrome	2	*CHD2* (1), *SMARCA2* (1)
Doose syndrome	1	*CHD2* (1)
Dravet syndrome	1	*CHD2* (1)
SE	2/32 (6.3%)	*CHD2* (1), *SMARCA2* (1)
Seizure‐free	20/29 (69.0%)	*CHD2* (13), *CHD3* (1), *ARID1B* (1), *SMARCA2* (4), *BPTF* (1)
History of febrile seizures	7/50 (14.0%)	*CHD2* (5), *CHD3* (1), *CHD4* (1)
Febrile seizures without epilepsy	4/50 (8.0%)	*CHD2* (2), *CHD3* (1), *CHD4* (1)
GDD/ID	38/50 (76.0%)	/
Learning difficulty	3/50 (6.0%)	*CHD2* (3)
ASD/autistic behavior	8/50 (16.0%)	*CHD2* (3), *CHD8* (2), *ARID1B* (2), *ARID2* (1)
ADHD/hyperactivity	6/50 (12.0%)	*CHD2* (5), *ARID1B* (1)
Tic disorder	1/50 (2.0%)	*CHD8* (1)
Microcephaly	7/50 (14.0%)	*ARID2* (1), *SMARCA2* (5), *BPTF* (1)
Short stature	9/50 (18.0%)	*SMARCA2* (7), *ARID1B* (1), *BPTF* (1)
Facial dysmorphism	16/50 (32.0%)	*CHD2* (1), *CHD3* (2), *CHD7* (1), *ARID1B* (2), *ARID2* (1), *SMARCA2* (8), *ACTB* (1)
Micropenis	2/50 (4.0%)	*CHD2* (2)
Cryptorchidism	3/50 (6.0%)	*ARID1B* (2), *SMARCA2* (1)
Single transverse palmar crease	5/50 (10.0%)	*CHD2* (2), *ARID1B* (2), *ACTB* (1)
Congenital heart disease	2/50 (4.0%)	*CHD7* (1), *ARID1B* (1)
Hearing impairment	1/50 (2.0%)	*CHD7* (1)
Normal cranial MRI	26/40 (65.0%)	/
Normal EEG	8/45 (17.8%)	/

### 3.4 Seizures and Treatment

**FIGURE 4:**
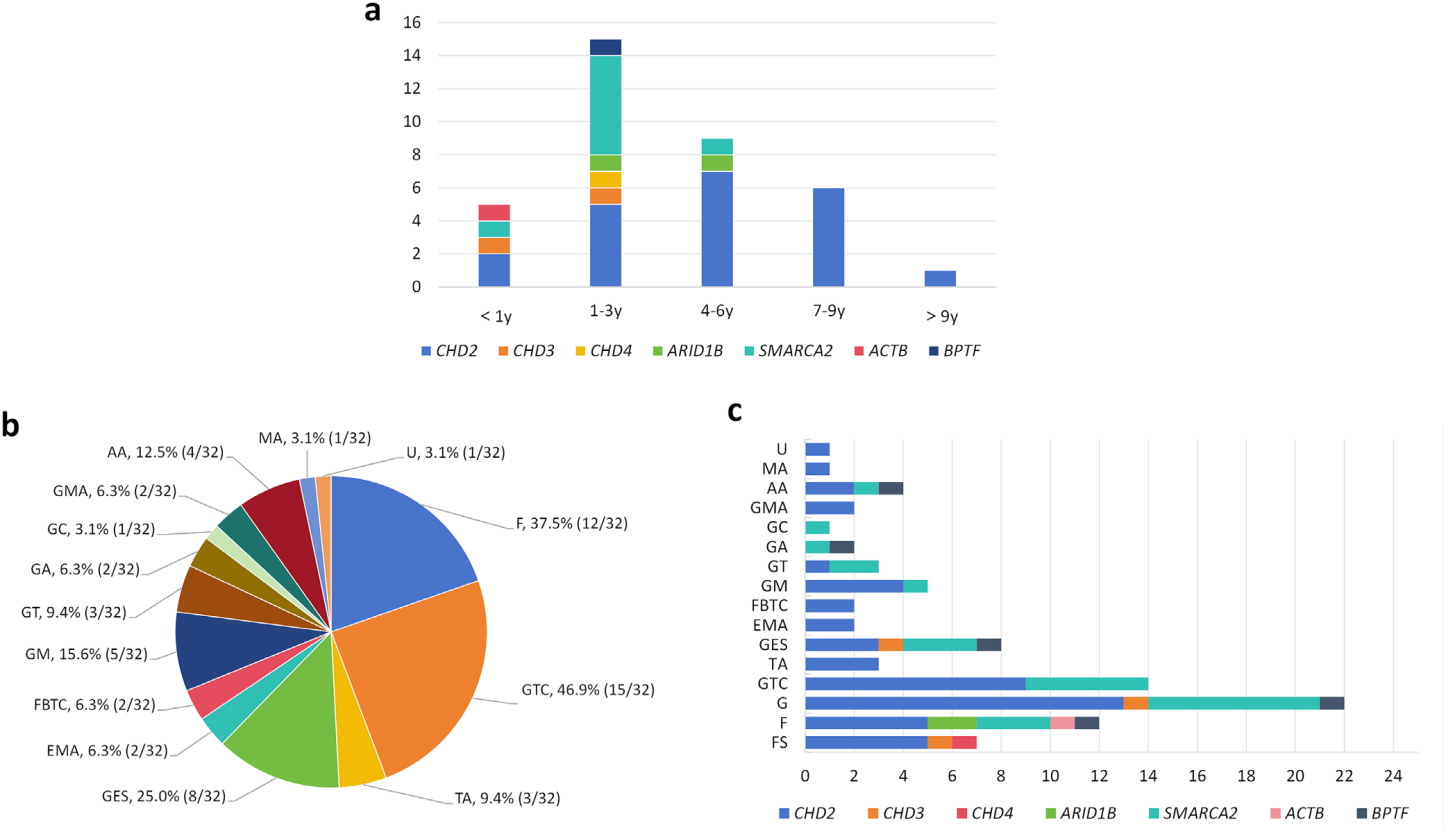
Semiology of CRC‐related epilepsy and FS. (a) Onset age of seizures in 36 patients with epilepsy or FS. (b) Specific seizure types involved in 32 patients with epilepsy. (c) Seizure types and FS distribution in 36 patients with epilepsy or FS. Seizures included: atypical absence seizures (AA), eyelid myoclonia with/without absence (EMA), focal seizures (F), focal‐to‐bilateral tonic–clonic seizures (FBTC), generalized seizures (G), generalized atonic seizures (GA), generalized clonic seizures (GC), generalized epileptic spasms (GES), generalized myoclonic seizures (GM), generalized myoclonic–atonic seizures (GMA), generalized tonic seizures (GT), generalized tonic–clonic seizures (GTC), typical absence seizures (TA), unclassified seizures (U).

In this cohort, 64.0% (32/50) of patients had epilepsy, and four patients (4/50, 8.0%) only had FS (Table 1). Three patients had a history of FS before afebrile seizures. The onset age of seizures ranged from 4 months after birth to 11 years old, and the median onset age was 2 years [IQR, 1–2]. Five patients (5/36, 13.9%) had their first seizure before 1 year old, and most patients (24/36, 66.7%) started at 1–6 years old. Seizures started after 6 years old in seven patients (7/36, 19.4%), and only one of them started at 11 years old. Seizures started at a large range of ages in patients with *CHD2* variants (Figure 4a), while the first seizure occurred before 7 years old in patients with other CRC variants.

For 32 patients with epilepsy, 19 patients (19/32, 59.4%) had generalized seizures, seven patients (7/32, 21.9%) had focal seizures, five patients (5/32, 15.6%) had both types of seizures, and the seizures of the remaining one patient (1/32, 3.1%) could not be classified. The specific types of seizures were various, including generalized tonic–clonic seizures (GTC) (15/32, 46.9%), generalized epileptic spasms (GES) (8/32, 25.0%), generalized myoclonic seizures (GM) (5/32, 15.6%), atypical absence seizures (AA) (4/32, 12.5%), generalized tonic seizures (GT) (3/32, 9.4%), typical absence seizures (TA) (3/32, 9.4%), focal‐to‐bilateral tonic–clonic seizure (FBTC) (2/32, 6.3%), generalized atonic seizures (GA) (2/32, 6.3%), eyelid myoclonia with/without absence (EMA) (2/32, 6.3%), generalized myoclonic–atonic seizures (GMA) (2/32, 6.3%) and generalized clonic seizures (GC) (1/32, 3.1%) (Figure 4b). Patients with variants in *CHD2*, *SMARCA2*, and *BPTF* had more than four kinds of seizure types, while patients with variants in other CRC genes had only one kind of seizure (Figure 4c). There were other characteristics of seizures observed. Heat‐sensitive seizures occurred in five patients (5/32, 15.6%), and four of them had *CHD2* variants. Three patients with *CHD2* variants had reflex seizures, including photosensitive and sound‐sensitive seizures. Seizures were induced by sunshine in one patient with a *CHD2* variant. One patient with a *CHD2* variant and one patient with an *SMARCA2* variant had status epilepticus (SE) (Table 1).

Fifty percent of patients took one or two antiseizure medications (ASMs) (Table 2). Nine patients (9/32, 28.1%) had monotherapy, and one patient had one ASM plus ketogenic diet (KD). Five patients took valproate, while four patients used levetiracetam. Only one patient with focal seizures selected oxcarbazepine as the first choice. The combination of two ASMs was conducted in six patients (6/32, 18.8%). One patient diagnosed with IESS used prednisone and vigabatrin. Three groups were used in the other five: valproate and topiramate, oxcarbazepine combined with valproate or levetiracetam. Sixteen patients (50%, 16/32) had three or more than three kinds of ASMs, of which one had add‐on KD, and one had vagal nerve stimulation (VNS). Valproate, levetiracetam, topiramate, lamotrigine, and clonazepam were frequently used ASMs. Three patients were lost to follow‐up by the last visit, and one patient with an *ARID1B* variant died during a seizure attack, which was not controlled with three kinds of ASMs. Twenty patients (20/29, 69.0%) were seizure‐free for more than 1 year (Table 2), and half of them needed three or more kinds of ASMs (10/20, 50.0%).

**TABLE 2:** Treatments of 32 patients with epilepsy in this study.

Case No.	Gene	All ASMs used	Current ASMs	Seizure‐free
1	*CHD2*	LEV	−	Yes
2	*CHD2*	VPA, TPM	TPM	Yes
3	*CHD2*	VPA	−	Yes
4	*CHD2*	LEV, CZP, VPA	LEV, CZP, VPA	Yes
6	*CHD2*	OXC, VPA	OXC, VPA	Yes
7	*CHD2*	VPA, KD	−	Yes
8	*CHD2*	VPA, LTG, CLB	VPA, LTG, CLB	No
9	*CHD2*	LEV	LEV	Yes
10	*CHD2*	OXC, LEV, VPA, TPM	LEV, VPA, TPM	No
11	*CHD2*	TPM, VPA, CZP, LTG, LEV, PER	VPA, CZP, PER	Yes
12	*CHD2*	VPA, LEV, LTG	VPA, LTG	Yes
13	*CHD2*	VPA, ACTH, TPM, CZP, LTG, LCM	VPA, CZP, LTG, LCM	No
14	*CHD2*	VPA	Lost to follow‐up	Uk
15	*CHD2*	LEV, TPM, CZP	TPM, CZP	Yes
16	*CHD2*	TPM, VPA	Lost to follow‐up	Uk
17	*CHD2*	VPA, LTG, CLB	VPA, LTG	Yes
19	*CHD2*	VPA, LEV, LTG	VPA, LEV, LTG	Yes
20	*CHD2*	VPA, LTG, ZSM, CZP	VPA, LTG, ZSM, CZP	Yes
21	*CHD2*	VPA	VPA	Yes
24	*CHD3*	Prednisone, VGB	VGB	Yes
32	*ARID1B*	VPA, CZP, PER	Died at seizures attack	No
35	*ARID1B*	LEV	LEV	Yes
41	*SMARCA2*	LEV	LEV	Yes
42	*SMARCA2*	VPA	VPA	No
43	*SMARCA2*	OXC, LEV	LEV	Yes
44	*SMARCA2*	VPA, LTG, ACTH, NZP	LTG	Yes
45	*SMARCA2*	ACTH, TPM, LTG	TPM	Yes
46	*SMARCA2*	VPA, LTG, VNS, LEV, NZP, TPM	LEV, NZP, TPM	No
47^a^	*SMARCA2*	TPM, LEV, LTG, VPA, CLB	−	No
48	*SMARCA2*	OXC, LEV	OXC, LEV	No
49	*ACTB*	OXC	OXC	No
50	*BPTF*	VPA, TPM, CZP → CLB, KD, ACTH	VPA, TPM	Yes

Abbreviations: −, not applicable; ACTH, adrenocorticotropic hormone; CBZ, carbamazepine; CLB, clobazam; CZP, clonazepam; KD, ketogenic diet; LCM, lacosamide; LEV, levetiracetam; LTG, lamotrigine; NZP, nitrazepam; OXC, oxcarbazepine; PER, perampanel; TPM, topiramate; Uk, unknown; VGB, vigabatrin; VNS, vagal nerve stimulation; VPA, valproate; ZSM, zonisamide.

^a^ The patient discontinued all ASMs without following medical advice.

### 3.5 Developmental Delay and Behavioral Problems

GDD/ID was presented in 76.0% (38/50) patients, and 18 of them (18/38, 47.4%) had severe to profound GDD/ID. Meanwhile, three patients with variants in *CHD2* only manifested as learning difficulties. Behavioral problems were also common. Sixteen percent (8/50) of patients had ASD or autistic behavior. Three patients had ADHD, and three patients had symptoms of hyperactivity (Table 1).

### 3.6 EEG and Cranial MRI

EEG recording was conducted in 45 patients at one or more times. Eight patients (17.8%) had normal EEG. Of the 37 patients with abnormal EEG, 89.5% (34/37) had interictal epileptiform discharges (IEDs), while the other three cases only displayed slow waves (Table 3). Different IEDs were itemized. Generalized or diffuse IEDs were the most common, presenting in 21 patients (21/33, 63.6%). Four patients had bilateral multifocal IEDs, and two patients had unilateral multifocal IEDs. Unilateral focal IEDs and hypsarrhythmia occurred in three patients, respectively. More than half of the patients (22/37, 59.5%) showed slow background activity, and 35.1% (13/37) of patients had slow waves on EEG, of which ten had generalized or multifocal slow waves, and three had focal slow waves. Specifically, seven patients with *CHD2* variants had photoparoxysmal responses (PPRs) or photoconvulsive responses (PCRs) induced by intermittent photic stimulation (IPS). Particularly worth mentioning is that discharges disappeared in 7 patients (7/33, 21.2%) during the follow‐up process (Table 3).

**TABLE 3:** EEG anomaly in 37 patients.

Characteristics	*N* (%)	Genes (No. of patients)
Slow background activity	22/37 (59.5%)	*CHD2* (8), *CHD3* (1), *ARID1B* (3), *SMARCA2* (8), *ACTB* (1), *BPTF* (1)
Slow waves	13/37 (35.1%)	*CHD2* (5), *ARID1B* (3), *SMARCA2* (4), *BPTF* (1)
Generalize/multifocal slow waves	10	*CHD2* (5), *SMARCA2* (3), *BPTF* (1), *ARID1B* (1)
Focal slow waves	3	*ARID1B* (2), *SMARCA2* (1)
Interictal epileptiform discharges (IEDs)	33/37 (89.2%)	
Generalized/diffuse IEDs	21/33 (63.6%)	*CHD2* (15), *SMARCA2* (5), *BPTF* (1)
Bilateral multifocal IEDs	4/33 (12.1%)	*CHD2* (3), *CHD7* (1)
Unilateral multifocal IEDs	2/33 (6.1%)	*CHD2* (1), *ATCB* (1)
Unilateral focal IEDs	3/33 (9.1%)	*ARID1B* (2), *SMARCA2* (1)
Hypsarrhythmia	3/33 (9.1%)	*CHD2* (1), *CHD3* (1), *SMARCA2* (1)
PPR/PCR induced by IPS	6/33 (18.2%)	*CHD2* (6)
Discharges disappeared	7/33 (21.2%)	*CHD2* (3), *CHD3* (1), *SMARCA2* (2), *BPTF* (1)

Abbreviations: IPS, intermittent photic stimulation; PCR, photoconvulsive responses; PPR, photoparoxysmal responses.

Forty patients had undergone cranial MRI, and the majority of them (26/40, 65.0%) were normal. The relatively common anomalies were nonspecific white matter lesions and ventriculomegaly, presented in four and three cases, respectively. Other abnormal findings, like Arnold‐Chiari malformation I, Dandy‐Walker malformation, hippocampal sclerosis, hippocampal swelling, and venous malformation, were less common (Table 4).

**TABLE 4:** MRI anomaly in 14 patients.

Characteristics	*N* (%)	Genes (No. of patients)
Arnold–Chiari malformation I	1/14 (7.1%)	*ACTB* (1)
Dandy–malformation	1/14 (7.1%)	*ARID1B* (1)
Hippocampal sclerosis	1/14 (7.1%)	*SMARCA2* (1)
Hippocampal swelling	1/14 (7.1%)	*CHD2* (1)
Nonspecific white matter lesions	4/14 (28.6%)	*CHD4* (1), *ARID1B* (1), *SMARCA2* (1), *BPTF* (1)
Ventriculomegaly	3/14 (21.4%)	*CHD2* (1), *CHD3* (1), *SMARCA2* (1)
Subarachnoid space enlargement	1/14 (7.1%)	*CHD3* (1)
Cyst	1/14 (7.1%)	*ARID1B* (1)
Hypomyelination	1/14 (7.1%)	*CHD3* (1)
Agenesis of corpus callosum	1/14 (7.1%)	*ACTB* (1)
Venous malformation	1/14 (7.1%)	*CHD8* (1)

### 3.7 Phenotype Comparisons Between the CHD and BAF Complexes

In our study, patients with variants in the CHD and BAF complexes composed the majority of the samples. To better show the distinction of these two complexes, comparisons of phenotype were conducted (Table 5). Only those phenotypes that existed in more than five patients were compared. Also, phenotypes that only presented in patients with variants in a single gene were not compared. Statistical results disclosed that the existence of epilepsy, seizure types, number of ASMs, and ASMs response were not different between the CHD and BAF groups. But patients in the CHD group were more likely to have heat‐sensitive seizures (FS included) (FDR = 0.048). Patients in the BAF group were more prone to present GDD/ID (FDR = 0.032), microcephaly (FDR = 0.009), facial dysmorphism (FDR = 0.030), and short stature (FDR < 0.001). The incidence of behavior problems, both ASD/autistic features and ADHD/hyperactivity, was not different between the two groups either. The EEG results and cranial MRI results were also compared. It turned out that patients with BAF complex variants were more likely to have slow background activity on EEG (FDR = 0.043).

**TABLE 5:** Phenotype comparisons between the CHD and BAF complexes.

Phenotypes	CHD complex	BAF complex	*p*	FDR (BH)^a^
Epilepsy	20/29 (69.0%)	11/20 (55.0%)	0.375	0.521
Generalized seizures	16/20 (80.0%)	7/11 (63.4%)	0.405	0.521
Focal seizures	5/20 (25.0%)	6/11 (54.5%)	0.132	0.264
Heat‐sensitive seizures^b^	11/29 (37.9%)	1/20 (5.0%)	0.016	**0.048**
≥ 3 ASMs	10/20 (50.0%)	5/11 (45.5%)	0.491	0.559
Seizure‐free	15/18 (83.3%)	5/10 (50.0%)	0.091	0.205
GDD/ID	20/29 (69.0%)	20/20 (100.0%)	0.007	**0.032**
Severe cognition impairment^c^	10/29 (34.5%)	10/20 (50.0%)	0.377	0.521
ASD/autistic behavior	5/29 (17.2%)	3/20 (15.0%)	1.000	1.000
ADHD/hyperactivity	5/29 (17.2%)	1/20 (5.0%)	0.379	0.521
Microcephaly	0/29 (0.0%)	7/20 (35.0%)	0.001	**0.009**
Facial dysmorphism	5/29 (17.2%)	12/20 (60.0%)	0.005	**0.030**
Short stature	0/29 (0.0%)	8/20 (40.0%)	< 0.001	**< 0.001**
Normal EEG	4/27 (14.8%)	4/17 (23.5%)	0.690	0.731
Slow background activity	9/27 (33.3%)	13/17 (76.5%)	0.012	**0.043**
Slow waves	5/27 (18.5%)	8/17 (47.1%)	0.087	0.205
Generalized/diffuse IEDs	15/27 (55.6%)	6/17 (35.3%)	0.228	0.410
Normal MRI	16/22 (72.7%)	10/17 (58.8%)	0.497	0.559

^a^ *p* values were adjusted for multiple testing using the Benjamini‐Hochberg (BH) method. Bold FDR values denote statistically significant differences (FDR < 0.05).

^b^ Patients with heat‐sensitive seizures and FS were all included.

^c^ Patients diagnosed as DEE with severe cognitive impairment and severe‐profound ID/GDD were included.

## 4 Discussion

Our study provided the genetic and phenotypic profile of CRC‐related neurological disorders in a Chinese pediatric cohort. A total of 50 cases were included, which will be vigorously used to improve the knowledge of CRCs. The role of CRCs in cancers has been thoroughly reviewed [17, 18]. The overview of CRCs in pediatric neurological disorders is relatively limited right now. Eleven years ago, Voge–Ciernia and A. Wood reviewed that the neuron‐specific BAF complex (nBAF) has roles in neuronal development and long‐term memory formation in the adult [2]. Recent studies have usually focused on the genotype and phenotype of specific genes encoding CRC, like *CHD2*, *SMARCA2*, and *ARID1B*. Thus, a clear and detailed report of CRC‐related pediatric neurological disorders is needed.

Twenty‐nine patients with variants in the CHD complex, 20 patients with variants in the BAF complex, and one patient with a variant in the ISWI complex comprised this group, revealing the proportion of different CRC genes in our center. To some extent, it also reflects the incidence rate and composition of CRC‐related neurological disorders in the Chinese population. In the literature, *CHD2* was the most frequently reported from different Chinese hospitals [19, 20, 21]. *SMARCA2* was the second most frequently reported one [22]. Variants in *ARID1B*, *ARID2*, *CHD4*, *CHD3*, and *CHD8* were reported in case reports [23, 24, 25, 26, 27]. This status is similar to our report. In our cohort, variants in *CHD2* had a proportion of 42.0%; variants in *SMARCA2* and *ARID1B* accounted for 34.0%; and variants in other genes accounted for the remaining 24.0%. Besides, there were 28 variants in this study that have not been reported in the literature or ClinVar before, expanding the genotype spectrum of those CRC genes. The clinical profile of CRC‐related neurological disorders can be revealed through our report.

Our findings demonstrated that the phenotypes in different CRC complexes are not homogeneous. GDD/ID is more frequent in the BAF group. Patients with BAF complex variants are also more prone to have microcephaly, facial dysmorphism, and short stature. Heat‐sensitive seizures, especially FS, are more likely to occur in patients with CHD complex variants. The underlying mechanism is not clear now. Different CRCs may influence the expression of different genes. For example, complete CHD2 loss led to impairment of inhibitory interneuron development and altered expression of genes involved in neurotransmission [28]. The BAF complex plays roles in neural stem cell generation, proliferation, migration, and differentiation, which are essential for neurodevelopment [29]. For instance, ARID1B was found to regulate Wnt/β‐catenin signaling, a typical signaling pathway in ID [30]. Epigenetics, such as methylation, may also contribute to the different phenotypes. Variants in *SMARCA2* can cause Nicolaides–Baraitser syndrome (NCBRS) and blepharophimosis intellectual disability syndrome (BIS). But different clinical characteristics exist between those two syndromes. The significantly different genome‐wide DNA methylation signatures between the NCBRS and BIS groups were supposed to cause the difference [31, 32]. Further exploration is in demand to disclose the mechanism behind the distinct phenotypes among different CRCs.

We reported 21 cases with *CHD2* variants, which also updated more information about *CHD2*‐related diseases, mainly the phenotype spectrum and treatments of *CHD2*‐related epilepsy. We found that FS and heat‐sensitive seizures can be a common phenotype of *CHD2*. There were two cases and two affected parents who had FS rather than epilepsy in our study. Meanwhile, three patients had a history of FS before afebrile seizures, and four patients had heat‐sensitive seizures. FS or febrile‐induced seizures have also been reported in several cases before [19, 33, 34]. In our work, clinical photosensitivity was less common, but PPR or PCR were recorded more frequently. PPR or PCR were monitored by EEG recording in 7 of 19 (36.8%) *CHD2*‐related epilepsies in this context, but only two of them had photosensitive seizures observed by guardians. Some of them were seizure‐free, and the EEG still showed PPR. Also, there was one patient (P17) who had seizures induced by sunshine, and the EEG did not show PPR.

Little is known about the seizure control rate of *CHD2*‐related epilepsy. It was notable that in our cohort, only four cases still had seizures, with a seizure‐free rate of 76.5% (13/17). For those who had seizures controlled, three cases had been off ASMs. Four of 13 well‐controlled patients only took one kind of ASM (Two had levetiracetam and two had valproate), and one patient with Doose syndrome became seizure‐free after valproate and add‐on KD. Most cases (7/13, 53.8%) had taken 2–3 kinds of ASMs. Less commonly, one patient had four kinds of ASMs, and one patient had tried six kinds of ASMs before becoming seizure‐free. Valproate, levetiracetam, and clonazepam/clobazam were frequently used ASMs, which had been reported in *CHD2*‐related seizures before [19, 20, 21]. Lamotrigine, perampanel, zonisamide, and lacosamide were used in several cases, too. Their effects need more data to estimate.

Except for the frequently reported genes, here we also reported genes that were rarely reported before, like *CHD4* and *BPTF*. In 2021, *CHD4* was reported to be a candidate causative gene of childhood idiopathic epilepsy with arrhythmia [23]. In this study, we enrolled one case with FS and arrhythmia having a de novo variant (p.K119Q) of *CHD4*, which was not reported before. FS and arrhythmia were common phenotypes in Liu's study [23]. A Color Doppler ultrasound was also done on our patient, and the result was normal. Our report further strengthens the clinical evidence for their findings. Patients with NDDs were reported to have variants in *BPTF* since 2017; epilepsy only occurred in several cases [6, 35]. Glinton et al. [35] reported 24 cases with variants in *BPTF*, and only three of them had clinical seizures, two of which were controlled by levetiracetam or valproate. One case with complex focal seizures needed VNS to control seizures. Our case had similar features of ID, microcephaly, and short stature, but more severe seizures. The first seizure occurred at 2 years old. The types of seizures included atonic, focal, epileptic spasm, and atypical absence. Valproate, topiramate, clonazepam (turned to clobazam), ACTH, and KD were tried during the course. Eventually, seizures stopped when KD was initiated. The EEG results changed from diffuse SWDs to discharge absence. Epilepsy caused by *BPTF* variants is rarely reported; therefore, general clinical patterns cannot be derived from this single case. The detailed presentation of this patient in our study is merely a summary of individual experience, intended to provide a personalized reference for clinicians in their diagnostic and therapeutic practices.

The present findings carry exact clinical implications for patients with variants in CRCs. First of all, we systematically summarized the clinical profiles of CRC‐related neurological disorders in a pediatric cohort. The relatively large sample size enables clinicians to appropriately order WES based on patients' phenotypic manifestations to facilitate definitive diagnosis. Next, we comprehensively illustrated the diverse clinical phenotypes for each gene, which supports physicians in conducting multidimensional clinical assessment for affected individuals. Then, we documented the ASMs administered to each patient with epilepsy, providing practical references for clinicians when selecting ASMs. Finally, we presented long‐term prognostic indicators (seizure control, EEG changes, severe cognitive impairment) that can facilitate physicians in communicating prognostic outcomes to patients and their families. For future research, an expanded sample size of multicenter cohorts is needed to verify those results in our study; mechanistic studies using cellular and animal models will help clarify core epigenetic regulatory pathways. Additionally, the development of targeted small‐molecule regulators and gene therapies represents a critical translational direction to advanced, precise treatment of CRC‐related neurological disorders.

## 5 Conclusions

We reported a cohort consisting of 50 pediatric patients with pathogenic or likely pathogenic variants in CRCs. In summary, CRC‐related neurological disorders are principally caused by variants in the CHD and BAF complexes. *CHD2*, *ARID1B*, and *SMARCA2* are frequently implicated genes. GDD/ID and epilepsy are the predominant phenotypes. Generalized seizures are the most common seizure type, and the types of seizures are variable. Seizures were controllable in nearly 70% of CRC‐related epilepsy. Phenotypic differences were observed between the CHD and BAF complexes.

## Author Contributions

Jing Peng contributed to the conceptualization and funding acquisition; Shimeng Chen contributed to the formal analysis, methodology, visualization, writing of the original draft, and funding acquisition; Fei Yin and Fang He contributed to the methodology and validation; Chen Chen contributed to the acquisition and interpretation of EEG data; Li Yang, Ciliu Zhang, Xiaolu Deng, Lifen Yang, and Fangyun Liu contributed to the acquisition of data. All authors read and approved the final manuscript.

## Funding

This work was supported by grants from the China Postdoctoral Science Foundation (General Program, No. 2025M771966), the Natural Science Foundation of Hunan Province (No. 2025JJ60695), and the National Natural Science Foundation of China (No. 82471488).

## Disclosure

The authors have nothing to report.

## Ethics Statement

The study design was approved by the Xiangya Hospital Ethics Committee in accordance with the Helsinki Declaration (202307148–2). Informed consent was obtained from the parents or guardians.

## Conflicts of Interest

The authors declare no conflicts of interest.

## Supporting information


**Table S1:** Detailed clinical and genetic information of 50 patients in this study.

## Data Availability

The data that support the findings of this study are available from the corresponding author upon reasonable request.
